# Integrated frailty and intrinsic capacity care model for community-dwelling older adults in Singapore: a rapid qualitative study of anticipated implementation barriers and enablers using the Consolidated Framework for Implementation Research and its Outcomes Addendum

**DOI:** 10.3389/frhs.2025.1563686

**Published:** 2025-04-24

**Authors:** Mimaika Luluina Ginting, Grace Sum, Sinead Zhen Wang, Yew Yoong Ding, Laura Tay

**Affiliations:** ^1^ Geriatric Education & Research Institute, Singapore, Singapore; ^2^ Family Medicine, SingHealth Polyclinics, Singapore, Singapore; ^3^ Department of Geriatric Medicine & Institute of Geriatrics and Active Ageing, Tan Tock Seng Hospital, Singapore, Singapore; ^4^ Lee Kong Chian School of Medicine, Nanyang Technological University, Singapore, Singapore; ^5^ Geriatric Medicine Department, Sengkang General Hospital, Singapore, Singapore

**Keywords:** intrinsic capacity, frailty, ICOPE, Consolidated Framework for Implementation Research, implementation research, enablers and barriers, Singapore

## Abstract

**Introduction:**

Older adults are at increased risk of experiencing multimorbidity and care dependency due to declines in their physiological reserves. Optimizing the intrinsic capacity and functional ability of individuals is important to enable healthy aging. We engaged potential implementers of an integrated, community-based model for frailty and intrinsic capacity care, adapted from the World Health Organization Integrated Care for Older People framework, to assess the anticipated barriers and enablers to implementation within Singapore's healthcare context.

**Methods:**

The updated Consolidated Framework for Implementation Research (CFIR) and its Outcomes Addendum was adopted as the conceptual framework. Qualitative data were collected through focus group discussions (FGDs). We used a rapid qualitative inquiry approach, incorporating a combination of Rapid Research, Evaluation and Appraisal Lab sheet, the Rapid Identification of Themes from Audio recordings, and mind-mapping techniques for data synthesis, analysis, and interpretation. The framework approach was applied to structure and explore the qualitative data for triangulation across FGDs.

**Results:**

Five FGDs were conducted with 22 potential implementers (doctors, nurses, physio/occupational therapists, and community partners) between July and August 2023. We identified 24 CFIR determinants covering five domains (innovation, outer setting, inner setting, individuals, and implementation process). Enablers included intersectoral collaboration (partnership and connections), trialability (innovation trialability), alignment with overarching goal (mission alignment), and removal of hurdles and sufficient support (tailoring strategies). Barriers included complexity (innovation complexity), affordability (innovation cost), tradeoffs (relative priority), synergy among multiple programs (compatibility), resource intensity (available resources), fragmented understanding of the care model across providers (communication), physical spaces' design (physical infrastructure), limited time and resources (innovation deliverers' opportunity), gaps in clients' capability (capability), and non-compliance (motivation). Policy contexts and directives (policies and laws), theoretical benefits (innovation evidence base), comprehensiveness and patient-centeredness (design), enhanced service access (relative advantage), proposed task allocation (work infrastructure), information access (information technology infrastructure), capability building (access to knowledge and information), innovation deliverers' capability, motivation, and accessibility (innovation recipients' opportunity) were both barriers and enablers.

**Discussion:**

The findings demonstrated agreement with the innovation and suggested implementation readiness at clinical and service levels. However, addressing key barriers and leveraging existing enablers are necessary for successful adoption and implementation.

## 1 Introduction

With increasing age, older adults experience physiological changes and vulnerability to chronic comorbidities and disabilities that culminate in care dependency (1, 2). Moreover, the reduction in physiological reserves contributes to frailty, a geriatric syndrome characterized by increased vulnerability to stressors and a higher risk of adverse health outcomes (2, 3). This calls for healthcare systems to improve coordinated strategies to deliver more integrated health and social care services capable of responding to diverse and complex care needs.

World Health Organization (WHO) defines healthy aging as the process of developing and maintaining functional ability, which enables wellbeing in old age (4). Intrinsic capacity (i.e., the composite of physical and mental capacities) is essential to healthy aging, as it represents the number of functional reserves that individuals can draw upon, which, alongside environmental factors, influence their functional ability. The assessment of intrinsic capacity and frailty can be considered complementary, representing a continuum from reserves through deficits in health. In 2017, the WHO published the Integrated Care for Older People (ICOPE) framework to guide care for older adults from a function-centered and person-centered perspective (5). The framework emphasizes the optimization of individual's intrinsic capacity and functional ability to promote healthy aging, prevent frailty, and reduce care dependency (5, 6). The innovation developed in this study, called INFINITY-ICOPE (Optimising Intrinsic Capacity for Functional INdependence and to Impede FrailTY in Older Adults: Adaptation of the WHO ICOPE for Healthy Ageing in Singapore), integrates frailty and intrinsic capacity into the entire ICOPE care pathway by employing a tiered approach to facilitate access to comprehensive geriatric assessment for frail, community-dwelling older adults in Singapore while monitoring intrinsic capacity trajectories for timely intervention.

Findings from the WHO readiness phase reported the feasibility of ICOPE implementation while emphasizing the value of local contextualization and co-design (7). A narrative review of early WHO ICOPE adopters and insights into its implementation reported mixed findings (8). The lessons learned might not be applicable in Singapore due to differences in implementation contexts. Implementing an evidence-based innovation requires an understanding of the context in which it will be implemented and often involves the need to change behavior. Many evidence-based healthcare innovations have not produced the anticipated health outcomes due to implementation failing to account for contextual factors (9). This study applied the updated Consolidated Framework for Implementation Research and its Outcome Addendum (CFIR-OA) to guide the assessment of anticipated contextual barriers and enablers, framed as CFIR determinants (across five domains: innovation, outer setting, inner setting, individuals, implementation process) that potentially influence implementation effectiveness (9, 10). The adoptability, or the likelihood that key decision-makers will decide to put the innovation in place or innovation deliverers will decide to deliver the innovation, was the anticipated implementation outcome that we focused on in this pre-implementation study. In addition, we also focused on understanding the CFIR determinants and a few antecedent assessments (acceptability, appropriateness, and feasibility) to predict the anticipated implementation outcome.

## 2 Materials and methods

### 2.1 Study aims and design

The 5-year INFINITY-ICOPE project uses a Hybrid Type 2 effectiveness-implementation design (11). This study reports on the pre-implementation phase conducted during the first year.

In this study, we aimed to identify the anticipated barriers and enablers to the delivery of the innovation, specifically focusing on its acceptability, appropriateness, feasibility, and adoptability. Acceptability was defined as the extent to which the innovation was perceived as agreeable or satisfactory to the stakeholders. Appropriateness was defined as the innovation fit to address frailty and intrinsic capacity deficits and its compatibility with the practice setting. Feasibility was defined as the extent to which the innovation can be successfully used or implemented within a given agency or setting. Finally, adoptability referred to as the intention, initial decision, or action to try or employ the innovation.

We adopted the rapid qualitative inquiry (RQI) approach (12). It is based on the concept of having intensive teamwork as part of the triangulation during the iterative process of data collection and analysis to allow a rapid preliminary understanding of a situation and nearly real-time sharing of emerging findings to stakeholders and identification of gaps before the fieldwork is completed (12). We incorporated the CFIR-OA as a guiding framework for data collection and analysis.

Our qualitative approach was anchored in pragmatism, allowing us to answer the research questions by choosing appropriate tools and techniques rather than being constricted within a specific tradition (13). This aligns with the rapid qualitative research conceptualized as a continuum, where different approaches could have different positions, with features borrowed from multiple approaches to answer the research questions (14). Ontologically, our approach generally fell within realism, where we recognized the importance of participants' interpretation in answering the research questions while acknowledging the existence of varied perspectives in understanding a complex phenomenon (15). We also believed in the importance of context in understanding the perspectives, aligning with the interpretivism paradigm, and this belief was reflected by the mix of deductive and inductive approaches across data collection, analysis, and interpretation phases (16).

This study was approved by the SingHealth Centralised Institution Review Board (CIRB) (Ref.: 2023/2058). The reporting of this study follows the Standards for Reporting Qualitative Research (SRQR) (Supplementary Table S1) (17).

### 2.2 Sampling

Sampling was purposive, stratified by professional role, and was based on participants' potential roles and involvement in the INFINITY-ICOPE implementation. We aimed for maximum variation to explore different perspectives from different implementer groups. Each FGD consisted of participants sharing the same potential role within the INFINITY-ICOPE care model but from varied implementation sites. This allowed us to observe the different contextual factors across implementation sites while allowing participants from various settings to interact and build a shared understanding and collective response on the topics. The different disciplines across participating organizations and settings included public and private primary care doctors, nurses, physiotherapists, occupational therapists, and staff of community sites/activity centers for older adults. We selected participants with varying intensities of involvement in community frailty identification, management, or complex care delivery in Singapore.

### 2.3 Innovation

INFINITY-ICOPE is a community-based, multi-disciplinary care model that aims to support and optimize functional ability through coordinated screening and management of frailty and intrinsic capacity deficits. The model targets community-dwelling older adults aged 60 years and above, including new or existing clients of community sites/activity centers serving older adults.

**Figure 1:**
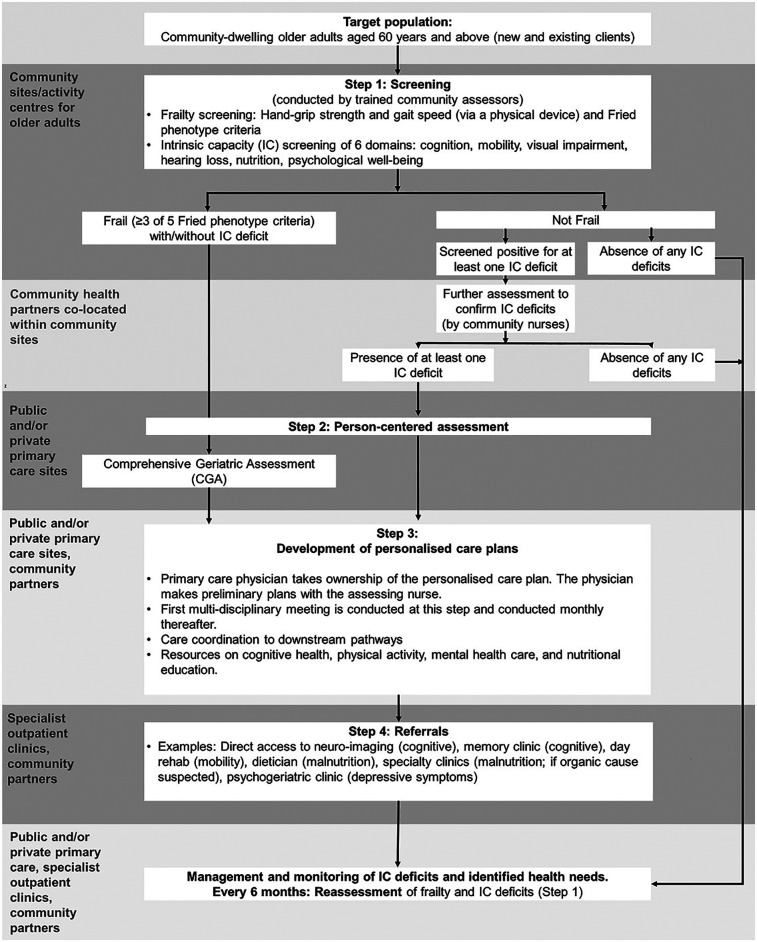
Patient journey diagram.

The INFINITY-ICOPE model includes five components: (i) frailty and intrinsic capacity screening at community sites/activity centers for older adults, (ii) clarification of potential intrinsic capacity deficits identified in screening, (iii) person-centered assessment at primary care sites, (iv) development of personalized care plans and multi-component interventions, and (v) referral pathway to specialized care. Physical frailty assessment is facilitated by an automated device that integrates gait speed and handgrip strength. A mHealth app has also been developed to support self-monitoring of intrinsic capacity and encourage self-management. Older adults who are not technologically savvy will receive access to face-to-face assessments and coaching. Multi-disciplinary team meetings are held monthly to review and coordinate care plans. Regular re-assessment of frailty and intrinsic capacity status is done every 6 months. Figure 1 shows the patient journey diagram.

### 2.4 Data collection

Focus group discussions (FGDs) were used to generate qualitative data. The FGD was complemented by describing the patient journey diagram to participants prior to inquiring about their perspectives.

We developed a semi-structured topic guide with questions driven by the concepts from the CFIR-OA framework (Supplementary Table S2). The guide included topics that assess pre-implementation acceptability, appropriateness, feasibility, and adoptability of the INFINITY-ICOPE model (9, 10). Each question was mapped to selected CFIR determinants to ensure that we probed for the most relevant information across the framework. We paired the CFIR determinants with the selected anticipated implementation outcome (i.e., adoptability) and antecedent assessments (i.e., acceptability, appropriateness, feasibility). The pairing was guided by the initial hypothesis of which CFIR determinants predicted the antecedent assessments and anticipated implementation outcomes. This *a priori* hypothesis informed our initial coding categories that comprise the codebook, which we used to deductively guide the coding process. A further iteration of the codebook was derived inductively from the data.

Data collection was staggered over an approximately 5-week period. Each FGD was facilitated by two health service and implementation science researchers: a trained qualitative researcher with a master's degree in public health and clinical background (MG) and a researcher with a doctorate in public health (GS). All FGDs were audio-recorded.

### 2.5 Data analysis

We utilized a combination of the Rapid Research, Evaluation and Appraisal Lab (RREAL) sheet, Rapid Identification of Themes from Audio (RITA) recordings, and mind-mapping techniques to facilitate rapid data synthesis and analysis (18–20). After each FGD, field notes were synthesized into a table-based matrix corresponding to the main topics and questions from the semi-structured FGD guide or RREAL sheet. The RREAL sheet served as a working document to organize the preliminary categories or topic areas. The note taker entered summaries of participants' answers into the RREAL sheet and noted instances where additional detail or timestamp was needed. The second team member then reviewed the RREAL sheet, listened to the audio recordings, and built upon the first team member's notes. Concurrently, RITA coding was conducted independently by the same team members. Both team members worked iteratively to refine the RREAL sheets. The following steps were included: (i) each member independently identified key themes directly from listening to audio recordings of 5-min segments, tagged them to the relevant constructs from the CFIR-OA, and coded for presence and valence (i.e., positive/enabler, negative/barrier, or neutral mentions or influence toward anticipated implementation outcome) (Supplementary Table S3); (ii) discussion and review of coding between the two team members; (iii) creation of mind maps by one team member to explore higher-order thematic relationships, patterns, and explanations in the data (Supplementary Figure S1); and (iv) discussion and review of mind maps between the two team members. The mind map was utilized as a visual tool that facilitated sense-making and categorization of data that informed the synthesis and narration of findings in the RREAL sheets. The preliminary topic areas were used as the main anchor of the initial mind maps. The associated interlinkages of constructs and topics were explored and constructed to refine the narration.

**Figure 2:**
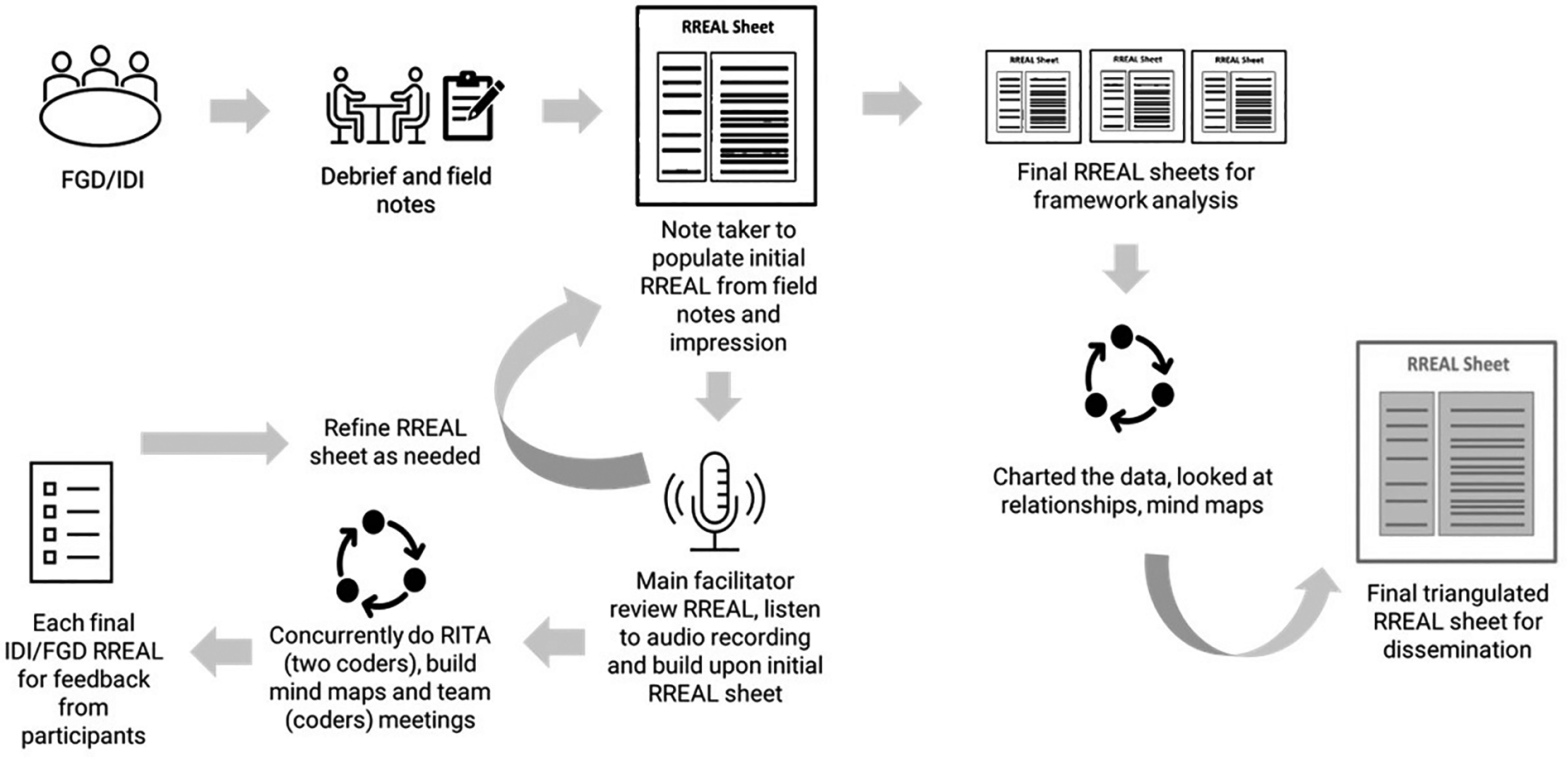
Methods and analytical approaches.

The study team then sought concurrence from participants on the synthesized RREAL sheet. We developed one RREAL sheet per FGD to allow comparisons across different sampling groups (Supplementary Data S1). A framework analysis approach was applied to further structure, organize, explore, and synthesize the qualitative data across different FGDs/RREAL sheets (21). Specifically, the steps of charting, mapping, and interpretation of data were executed. The topic areas within the respective RREAL sheets were plotted on a thematic chart. Each FGD was treated as the unit of analysis and allocated a row in the matrix, while each topic area was displayed in a separate column. The data from each thematic chart were further synthesized to reflect the key points while preserving the context for interpretation. Mapping and interpretation were done by comparing experiences across different units of analysis. A mind map was utilized to facilitate final data synthesis and narration. Ultimately, each RREAL sheet was collated into a single triangulated RREAL sheet. Microsoft Excel and NVivo 12 (QSR International, Doncaster, Australia) were used for data management. Figure 2 describes the analytical process.

We selected two FGDs for complete transcription to be coded conventionally to check for consistency of codes or themes generated between the rapid and conventional approaches.

## 3 Results

### 3.1 Characteristics of participants

Five FGDs were conducted with 22 potential implementers between July and August 2023 (40.9% doctors, 22.7% nurses, 18.2% physio/occupational therapists, and 18.2% community center staff; the number of years working as a healthcare provider ranged from less than a year to 20 years) (Table 1).

**Table 1:** Characteristics of participants.

Stakeholder group	Description of participants' profiles	Number of FGDs	Number of participants
Doctors	• Junior and mid-senior levels with a range of experience from less than a year to 20 years • Some had been involved in delivering complex interventions • From public primary care clinics, private practices, and community care organizations • Potential involvement in Steps 2, 3, and 4^a^	2	FGD 1, *n* = 4
			FGD 2, *n* = 5
Nurses	• Mid-senior level with 3–12 years of experience in primary care clinic • Some had been involved in delivering complex interventions, geriatric, and community care • Potential involvement in Steps 1, 2, 3, and 4^a^	1	*n* = 5
Physio/occupational therapists	• Junior to mid-senior level with 2–8 years of experience • From community care organizations • Potential involvement in Steps 3 and 4^a^	1	*n* = 4
Community center staff	• Junior and mid-senior levels with a range of experience from less than a year to 17 years of experience in working with older adults • Mixed roles, with some involved in day-to-day operations and others as decision-makers (planning, supervisory role) • Potential involvement in Step 1^a^	1	*n* = 4
Total		5	*n* = 22

^a^ Step 1: screening; Step 2: person-centered assessment; Step 3: development of personalized care plans; Step 4: referrals.

**Figure 3:**
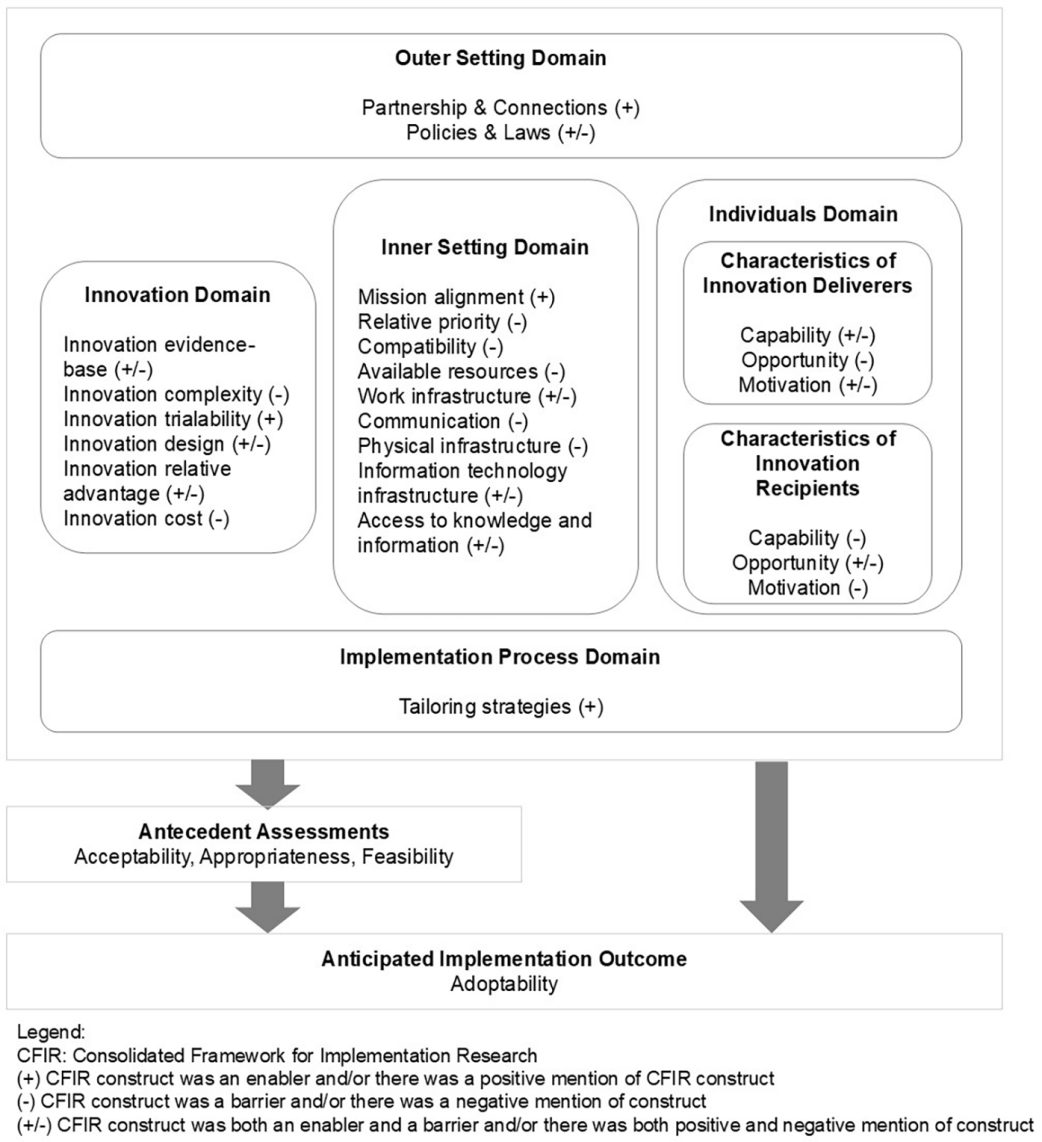
CFIR determinants, antecedent assessments, and anticipated implementation outcomes.

We identified 24 CFIR determinants that potentially facilitate or hinder implementation (4 were enablers only, 10 were barriers only, and 10 were both enablers and barriers), covering five domains (Figure 3).

The determinants captured setting- and individual-level enablers and barriers influencing the antecedent assessments (acceptability, appropriateness, and feasibility) and anticipated implementation outcome (adoptability). In addition, the antecedent assessments also contributed to the likelihood of successful adoption or adoptability.

### 3.2 Perspective on the innovation

#### 3.2.1 Innovation evidence base

The evidence supporting the potential benefit of INFINITY-ICOPE was deemed to be robust, with strong theory and recommendations from the WHO (22). It was considered appropriate for patients with frailty, intrinsic capacity deficits, and complex needs due to its comprehensive design that targets multiple domains (cognition, mobility, nutrition, vision, hearing, and mood) and its multi-disciplinary approach. However, there was expressed uncertainty regarding its effectiveness in eventually impacting patients' outcomes, given the multi-component nature of the interventions targeting multiple conditions embedded within the care model and the potential complexity and high resources needed in operationalizing it.

#### 3.2.2 Innovation complexity and trialability

The innovation was perceived as potentially complex due to the possibility of patients having different permutations of frailty levels and intrinsic capacity deficits, resulting in different combinations of downstream referrals and care interventions and requiring the involvement of multiple units/teams in care delivery. These different pathways and coordination needs were expected to lead to high administrative burdens and operational challenges. The ability to pilot test the innovation was deemed important to assess its appropriateness in the practice setting.

#### 3.2.3 Innovation design and relative advantage

As the innovation delivered integrated care for multi-domain problems, it was deemed relatively better than existing programs in participants' practice settings. Another anticipated advantage was convenience, from the proximity of older adults' residences to the screening location.

The innovation design that promoted patient-centeredness with a tailored care plan and sustained relationship with a dedicated care team was considered beneficial and aligned with older adults' preferences. In addition, the screening device was expected to nudge older adults' active involvement by increasing their health awareness with visual displays of their performance measurements (e.g., gait speed, grip strength). Participants suggested alternatives to using the mHealth app for self-screening, e.g., physical forms (with pictorial diagrams for illiterate persons) or a communal kiosk for self-screening at strategic locations. They also proposed for the mHealth app to include educational resources, reminders (appointments, intrinsic capacity screening), and appointment calendar functions.

An anticipated challenge was synergizing processes and different screening instruments across multiple programs within the practice setting. Without streamlining, multiple healthcare programs were anticipated to result in screening fatigue and reduced buy-in.

### 3.3 Characteristics of the inner setting

#### 3.3.1 Mission alignment and relative priority

Participants acknowledged that INFINITY-ICOPE’s emphasis on the importance of preventive health and the provision of appropriate, cost-effective care within the community for complex needs and frailty was aligned with population health objectives and the overarching commitments and goals to care for older adults within the community. However, different sectors expressed a different relative priority over the importance of the preventive and community-based approach. There was resistance from the private practices to accept the innovation, as they tend to focus on curative health and seldom need to work with a multi-disciplinary team or across different service providers. Community organizations also shared that they prioritized running their existing programs over innovation because of their scarce workforce.

#### 3.3.2 Incompatibility with practice capacity and work infrastructure

First, a mismatch between the anticipated high patient load resulting from the preventive screening approach and the capacity of practice settings was expected, which could lead to operational challenges and overwhelm providers. This was in addition to the expected challenges from the high turnover rate among clinicians and nurses and lack of motivation for training in geriatric care. Nurses shared the high demand and intensity of their workload, particularly related to complex cases and comprehensive assessment within a constricted time frame, while juggling multiple roles and longer working hours. Community partners shared their concerns about the potential high administrative demand and additional administrative work related to the study. Participants shared the need to assess and review the efficiency of the work infrastructure and task allocation within the multi-disciplinary team dynamic. Further, participants also viewed that the intensity and comprehensiveness of care should not be blanketed across all frailty levels. A suggestion was to ring-fence resources for the highest-need population segment or those who are very frail initially while directing others to existing services.

Second, a mismatch between the current roles of participants and the roles they need to play for the innovation was highlighted. Clinicians in the private sector felt that their involvement might be more appropriate for new patients who do not yet have a designated primary care provider. There was a perceived redundancy in referring patients with chronic conditions already under the care of multiple healthcare providers to a new primary care provider under this program.

#### 3.3.3 Communication

From participants' prior experience with implementing complex care models, there was often a fragmented understanding of care models across various providers. A key consequence observed by participants was that patients received different information about the health program and their care plans across touchpoints with multiple health and social service providers. This was perceived to diminish effective care and reduce patients' motivation to follow up on interventions.

#### 3.3.4 Physical and information technology infrastructure

Participants discussed the challenges of having limited space available that might contribute toward the problem of limited appointment timeslots. In addition, different healthcare providers were often physically located on a different story within the same building or were at different locations. Participants conveyed that this resulted in patients perceiving services as fragmented and inconvenient.

Participants expressed the need for timely access to comprehensive information about patients' needs and the services rendered to them within and outside the innovation. An integrated digital platform that allowed timely and seamless data and information-sharing across multiple settings was expected to help. However, there was perceived uncertainty on its feasibility due to the anticipated high administrative burden of the data integration effort. A possible alternative solution suggested was having a dedicated point of contact person to communicate about patients' needs and information.

### 3.4 Characteristics of individuals

#### 3.4.1 Innovation deliverer: capability, opportunity, and motivation

There were differences in the level of understanding across participants about the innovation, its intention, potential benefits, and how different components (or providers) fit into the overall care model, which led to a feeling of uncertainty about the unknown consequences of implementing it or being part of its delivery. Clinicians had more knowledge and ability to understand the intention and theory behind the innovation.

Most of the healthcare providers had gaps in knowledge about frailty and intrinsic capacity and in skills and experience of delivering geriatric care, which might lead to anxiety and lack of confidence in care delivery. The inability to speak multiple languages, including dialects, was also a barrier.

Participants viewed that it would be easier for providers to adapt their workflows if they had prior experience implementing community-based health programs similar to this innovation. The implementation would need to consider providing intensive training and direct one-to-one supervision by the implementation lead or experienced early adopters over an extended period. Another suggested possibility was that nurses could be empowered to deliver some aspects of chronic care and initiate referrals for a clearly defined sub-group of less complex patients (e.g., vision and hearing) to facilitate implementation.

Clear guidelines and protocols to navigate the care pathways were also deemed important, including criteria and feedback loops, mechanisms for monitoring and evaluation, and access to additional services (e.g., support for activities of daily living dependencies, day care services). There was expressed uncertainty on the feasibility of having written protocols due to many permutations in the care pathway.

The innovation's potential benefits for older adults served as an intrinsic motivator for implementers to embark on its implementation. An extrinsic factor conveyed was having incentives that are commensurate with delivering this resource-intensive model. Appropriate financial incentives were perceived to aid in cost efficiency, ensure sufficient profit margins, and provide remuneration compatible with the workload. The possibility of non-compliance and dropouts among older adults contributed to reluctance toward adoption, as there was a perceived wasted effort and resources from the comprehensive assessment and tedious administrative processes when patients do not adhere to care plans.

#### 3.4.2 Innovation recipient: capability, opportunity, and motivation

Participants noted that the care model emphasizes a preventive approach, promoting proactive health-seeking behavior, self-management, and adherence to follow-up plans. However, this was seen as challenging, given the characteristics of the current older adults aged 60 and above in Singapore, who often exhibit poor health literacy, passive health-seeking behaviors, and lack of self-management with a “quick-fix” mentality. In addition, the screening strategy that taps into the community centers' client network was perceived to be able to potentially miss older adults who were non-participants of these centers, have more unmet needs, and were frailer.

Gaps in potential client capability were also expected, such as difficulties in using technology, difficulties in going to multiple places for those with mobility issues or without caregiver support, difficulties in navigating the care pathway independently, and inability to afford the out-of-pocket costs.

Assigning program-specific befrienders or wellbeing coordinators to build rapport and journey with patients and understand patients' needs and priorities at different stages of their lives was deemed important in planning relevant care for patients and facilitating patients' compliance. Early communication about the innovation to gauge the patients' willingness to attend follow-ups, patient/caregiver education, and monetary incentives were viewed as enablers. Further, a multi-disciplinary team conducting collaborative assessments for patients in one sitting was suggested to tackle screening fatigue and dropouts from multiple appointments.

### 3.5 Characteristics of the outer settings

#### 3.5.1 Partnership and connections

Intersectoral partnership and collaboration with shared resources and effective resource allocation were deemed essential to support implementation. The public setting was perceived to be better positioned to take on this innovation because it was supported by a dedicated team with protected time and access to other supporting services (e.g., medical social workers, care managers, pharmacists).

#### 3.5.2 Policies and laws

Embarking on a new care model was perceived as demanding due to the high administrative burden it entails, and the fluctuating government directives at the primary care level further compounded the difficult situation. Finding synergy with wider policy directives was deemed necessary to facilitate implementation and improve buy-in from the target population. Participants noted the potential opportunity to leverage the recent national initiative in Singapore on preventing chronic conditions, where individuals were under the care of a primary care doctor of their choice and there was also a creation of personalized health plans. Instead of only involving a small number of primary care doctors in the implementation of INFINITY-ICOPE, participants anticipated benefits if there was a broader involvement of other primary care physicians. This was anticipated to be beneficial because of the stronger existing rapport between providers and patients, which promotes adherence. There would also be an anticipated reduction in workload.

### 3.6 Implementation process

#### 3.6.1 Tailoring strategies

Addressing barriers and leveraging on enablers were identified as keys to the likelihood of implementers deciding to adopt or implement INFINITY-ICOPE. Tailoring strategies and adaptations to fit the context in the practice setting and characteristics of individuals potentially involved in implementation were deemed vital in the successful adoption of innovation.

### 3.7 Relationships between CFIR-identified determinants, antecedent assessments, and anticipated outcome

We explored the relationships between the CFIR-identified determinants and domains identified within the key themes, as well as their relations with the antecedent assessments and anticipated outcomes (Table 2). The innovation domain was the predominant factor linked to acceptability, whereas the inner setting domain occupied the majority of determinants linked to appropriateness and feasibility. Finally, adoptability was mostly related to determinants from the individual domain.

**Table 2:** CFIR-identified barriers and enablers, antecedent assessments, and anticipated implementation outcomes.

Antecedent assessment/anticipated implementation outcome	Key theme	CFIR-identified determinant (barrier/enabler)^a^	CFIR domain
Antecedent assessment: Acceptability	Innovation complexity	Innovation complexity (−)	Innovation
	Resource intensity of care model	Available resources (−)	Inner setting
	Changing policy contexts and directives	Policies and Laws (−)	Outer setting
	Incompatibility with older adults' characteristics and needs	Innovation recipient: capability (−)	Individuals
		Innovation recipient: opportunity (−)	Individuals
	Lack of understanding of the care model and uncertainty about the unknown	Innovation deliverer: capability (−)	Individuals
	Innovation evidence base	Innovation evidence base (+)	Innovation
	Attention to patients' values and preferences	Innovation design (+)	Innovation
	Relative advantage of innovation	Innovation relative advantage (+)	Innovation
	Alignment with priorities	Mission alignment (+)	Inner setting
Antecedent assessment: Appropriateness	Uncertainty on effectiveness	Innovation complexity (−)	Innovation
		Innovation evidence base (−)	Innovation
	Theoretically sound care model	Innovation evidence base (+)	Innovation
	Mismatch between the anticipated patient load and the capacity of the practice	Compatibility (−)	Inner setting
		Available resources (−)	Inner setting
	Mismatch between the current roles of healthcare providers and community partners and the roles they need to play for innovation	Compatibility (−)	Inner setting
		Structural characteristics: work infrastructure (−)	Inner setting
	Lack of synergy among multiple programs in practice	Compatibility (−)	Inner setting
	Mission alignment	Mission alignment (+)	Inner setting
	Innovation trialability	Innovation trialability (+)	Innovation
	Work infrastructure and efficiency of task allocation	Structural characteristics: work infrastructure (+)	Inner setting
Antecedent assessment: Feasibility	Limited resources available to meet the demand of the care model	Available resources (−)	Inner setting
		Innovation deliverers: opportunity (−)	Individuals
		Structural characteristic: work infrastructure (−)	Inner setting
	Intersectoral partnership and collaboration with shared resources and effective resource allocation	Partnership and connections (+)	Outer setting
	Synergy with wider policy directives	Policies and laws (+)	Outer setting
	Gaps in the capability of implementers	Innovation deliverer: capability (−)	Individuals
	Targeted capability building	Access to knowledge and information (+)	Inner setting
	Gaps in clients' capability	Innovation recipient: capability (−)	Individuals
		Innovation design (−)	Innovation
		Innovation cost (−)	Innovation
	Fragmented understanding of the overall care model across providers	Communication (−)	Inner setting
		Innovation deliverer: capability (−)	Individuals
		Access to knowledge and information (−)	Inner setting
	Infrastructure hinders processes and effective care delivery	Structural characteristics: information and technology infrastructure (−)	Inner setting
		Structural characteristics: physical infrastructure (−)	Inner setting
	Timely access to comprehensive information on patients	Structural characteristics: information and technology infrastructure (+)	Inner setting
*Anticipated implementation outcome:* Adoptability	Relative priority and tradeoffs	Relative priority (−)	Inner setting
		Innovation relative advantage (−)	Innovation
	Demotivated from non-compliance and dropouts	Innovation deliverer: motivation (−)	Individuals
		Innovation recipient: motivation (−)	Individuals
	Removal of hurdles in implementation processes and sufficient support for resources	Tailoring strategies (+)	Implementation process
	Incentive systems	Innovation deliverer: motivation (+)	Individuals
	Belief in the potential benefit	Innovation deliverer: motivation (+)	Individuals
	Familiarity with similar programs	Innovation deliverer: capability (+)	Individuals
	Affordability and accessibility of the program	Innovation cost (−)	Innovation
		Innovation recipient: opportunity (+)	Individuals

^a^ (+) denotes an enabler and/or positive mention of the determinant; (−) denotes a barrier and/or negative mention of the determinant.

## 4 Discussion

Using RQI and guided by CFIR-OA, we engaged potential implementers prior to implementing INFINITY-ICOPE in Singapore to explore their perspectives on the anticipated barriers and enablers of the innovation's delivery, specifically focusing on its acceptability, appropriateness, feasibility, and adoptability. Enablers included intersectoral collaboration (partnership and connections), trialability (innovation trialability), alignment with overarching goal (mission alignment), and removal of hurdles and sufficient support (tailoring strategies). Barriers included complexity (innovation complexity), affordability (innovation cost), tradeoffs (relative priority), lack of synergy among multiple programs (compatibility), resource intensity (available resources), fragmented understanding of care model across providers (communication), limitation in physical spaces' design (physical infrastructure), limited time and resources (innovation deliverers' opportunity), gaps in clients' capability (capability), and non-compliance (motivation). Policy contexts and directives (policies and laws), theoretical benefits (innovation evidence base), comprehensiveness and patient-centeredness design (innovation design), enhanced service access (innovation relative advantage), proposed task allocation (work infrastructure), information access (information technology infrastructure), capability building (access to knowledge and information), innovation deliverers' capability, motivation, and accessibility (innovation recipients' opportunity) were both enablers and barriers. This study contributes to the broad literature on complex community-based interventions that include frailty and intrinsic capacity identification, comprehensive geriatric assessment, multi-disciplinary individualized care plans, downstream care interventions, and coordination of health services. Some of our findings are consistent with the WHO ICOPE “ready phase” report published in 2022 (7). The report described positive attitudes from healthcare workers toward the importance of person-centered integrated care to optimize intrinsic capacity and functional ability, with proactive engagement of older adults as the main key enabler across all ICOPE steps. Similarly, our study showed participants' agreement with the INFINITY-ICOPE design, which promoted person-centeredness by targeting multiple care domains delivered by a multi-disciplinary team. However, the WHO report also highlighted that the delivery of integrated care was complex and time- and resource-intensive, which required appropriate workforce capacity-building and enabling service delivery environments for successful implementation, echoing the potential barriers of innovation complexity, settings' capacity, and implementers' capability elicited in this study. The studies included in the report featured a mixed sample of those who had experienced the ICOPE pilot implementation and those who had not to inform further scale-up, whereas our study mainly focused on the perspectives of potential implementers prior to their direct experience. Notably, our study identified potential barriers and enablers that are contextualized to Singapore's primary and community-based healthcare system, contributing to the knowledge on the implementation and adaptation of the WHO ICOPE framework for large-scale community programs targeting older adults in regions with similar implementation contexts. Findings from this study will guide the mapping, selection, specifying, and development of implementation strategies to mitigate CFIR-identified contextual barriers and increase the likelihood of adoption and successful implementation in Singapore.

In this study, the acceptability of the innovation was largely facilitated by stakeholders' perspectives about the innovation, as reflected by the CFIR-identified determinants from the innovation domain (innovation evidence base, design, and relative advantage) (Table 2). It was attributed to the perception of the theoretical benefits and evidence supporting the care model, its comprehensiveness nature, enhanced availability and accessibility of specialized services, and its emphasis on patient-centered care. Our study suggested that perception here was largely theoretical or cognitively based. There was uncertainty about the innovation's effectiveness in eventually impacting patient outcomes due to the complexity of the innovation and resource intensity, which are linked to the perceived appropriateness of the innovation in addressing the issues of frailty and intrinsic capacity deficits. These findings partly align with the micro-survey of implementation readiness among 29 WHO member states, whereby there was a positive attitude toward the ICOPE approach and agreement on the importance of person-centered integrated care in optimizing older adults' functional ability (7). While the survey reported similar barriers to our study on workforce capacity and innovation complexity, our study elaborated more on the plausible link between these CFIR-identified barriers and the antecedent assessments (acceptability and appropriateness) and gave insights relevant to the local context. Acceptability and appropriateness were largely impeded by barriers from the individuals domain (limitation in the capability of potential implementers and clients and mismatch with the characteristics and needs of older adults in Singapore) and characteristics of the inner setting (mismatch with the practice capacity and work infrastructures), respectively. Our study suggests that the ability to test the innovation on a small scale, or trialability, was a key enabler in understanding the appropriateness of the innovation. This finding aligns with evidence showing trialability as an enabler to the adoption and assimilation of innovation in practice settings by allowing individuals to observe processes and evaluate potential results before making a long-term commitment to it (23, 24).

The literature suggests that the scale of adoption should be tailored according to the capacity of each practice setting (8). Globally, there have been variations in the adoption of the WHO ICOPE framework, with some of the early adopters implementing it using a more resource-efficient method by mapping their pre-existing data to examine the prevalence of positive cases as part of feasibility assessments, some applying the framework partially, and others progressing into full-scale implementation (8). Early adopters with the latter approach appeared to have more capacity to conduct large-scale programs (25). Similarly, in our study, practice capacity, capability of healthcare providers, and care coordination across providers involved in the program were the key components identified for the successful delivery of the program. With limited resources and capabilities available, it was deemed challenging to aim for large-scale implementation and sustainment. Implementation planning might benefit from mapping the existing resources within the health system and optimizing them by synergizing similar initiatives across all levels.

The nature of the resource-intensive innovation requires effective resource allocation planning. With active case-finding and screening initiatives, there should be sufficient workforce available for care delivery. Other resources are also needed, such as funding and infrastructure. For local practices that are more accustomed to seeing acute cases or providing activities related to social needs for older adults, implementing or partnering to implement the program was seen to involve tradeoffs, as it required allocating resources that could have otherwise been used for their existing programs. The care model also requires primary care physicians to own the care plan, enact the downstream care pathways, and coordinate care across different providers. This is challenging within the current primary care landscape in Singapore, where private general practitioners (GPs) deliver the majority of primary care services in Singapore. However, most operate as solo practices, with only a few practicing multi-disciplinary team-based care (26–28). On the other hand, primary care clinics operated by the public sector, or polyclinics, have access to multi-domain services, including nursing, medical social worker, health education, diagnostic, and pharmacy services; however, these clinics are currently strained by high patient volumes and a higher percentage of chronic care patients with complex conditions (27–29). More recently, in Singapore, primary care transformation has initiated efforts to expand the capabilities and capacities of primary care to deliver team-based care in the community, including reforms in primary healthcare financing, development of integrated care models for complex care in primary and community settings, and the enhancement of primary care networks among private GPs through funding and administrative support (28, 30–34). In addition, our findings emphasize the potential enabler of a public–private partnership as an essential pillar in scaling up team-based care and optimizing resource allocation.

A large-scale ICOPE implementation in the Occitania region highlighted the use of digital technology as a key enabler for care planning and timely follow-up (25). However, local adaptations are needed to address potential challenges related to accessibility, interoperability, integrity, data governance, cybersecurity, and usability. Similarly, findings from our study highlighted necessary adaptations and design flexibility to the proposed technology used in the innovation to fit the needs and characteristics of older adults in Singapore. The same study also reported challenges in implementing the downstream coordination and actualization of personalized care plans due to communication issues between stakeholders and older adults (25). Similar to our study, improving communication was perceived as an enabler of effective care coordination and delivery across different disciplines and settings. Suggestions included information integration via technology, systems infrastructure, or designated care coordinator.

Synergistic transformation and support from all levels of health systems, including the individual (micro) level, the service/organizational (meso) level, and the system (macro) level, are necessary to facilitate implementation (35). Implementation readiness needs to occur at all three levels, indicated by the acceptability and feasibility of the approach in the current practice, service capacity to respond to care needs, and system capacity to support integrated care. Our study suggests micro- and meso-level readiness, but barriers must be addressed. We did not specifically explore macro-level readiness in this study. However, a recent policy directive in Singapore indicates the commitment to support the integration of health and social care and adopt a whole-of-society response to optimize intrinsic capacity and functional ability to achieve healthy aging and prevent frailty, suggesting macro-level readiness (34).

Many theories model factors or attributes that shape health behavior at different levels, i.e., individual, interpersonal, community, and group models of behavior changes (36). Motivation or intention to perform a behavior is assumed to be the most crucial determinant of individual behavior in some models, with attitude, perceived norm, and personal agency as its direct influences (37). The interlinks and dynamics between these determinants in determining intention may vary for different behaviors and different populations. In our study, the intention to adopt the INFINITY-ICOPE project seemed to be influenced by both intrinsic motivating factors, like benefits to older adults and familiarity with the processes, and extrinsic motivating factors, like appropriate remuneration and cost efficiency. Other intrinsic factors included beliefs about the negative outcomes or attributes related to performing the behavior, such as patients' non-compliance and dropouts, the complexity of processes, and barriers in workforce capacity. Evidently, although the potential implementers agreed on the theoretical benefit of the potential innovation outcome, the motivation to adopt was primarily influenced by their attitude toward the perceived consequences attributed to the implementation processes, their perceived self-efficacy, and external constraints influencing the implementation outcome. Although this suggests a potential behavioral mechanism enacting adoption, we did not systematically embed the individual behavioral constructs while investigating the data and might not have captured all determinants comprehensively. Personal agency, defined as an individual's sense of ownership of control over performing, or planning to perform, a behavior, was proposed by the Institute of Medicine as a major factor influencing behavioral intention control (37). This construct consists of self-efficacy and perceived control. Based on this theory, addressing barriers related to individuals' confidence in the ability to perform the behavior (self-efficacy) and their perception of the degree of difficulties in performing the behavior due to various external/environmental factors (perceived control) may greatly influence motivation.

Within the CFIR framework, motivation and behavior are explored within the individual characteristics sub-domain. There was feedback highlighting challenges in using the CFIR constructs to understand barriers and enablers related to individuals, which limits the framework’s potential to understand individual's behaviors and identify the type of interventions most likely to be effective (9). The updated CFIR has attempted to restructure its individual-level domains to specify roles and introduce a characteristics sub-domain that includes constructs from behavioral theory, such as capability, opportunity, and motivation (9, 38). Despite the attempt to include individual-level characteristics as part of the determinants, we found that complementing CFIR with additional constructs from other theories, models, and frameworks, for instance, behavior change theories, might be beneficial when the implementation strategies specifically target the individual-level barriers and enablers. Mapping the constructs under the individual characteristics sub-domain to other relevant behavioral theories, e.g., the Theoretical Domain Framework or the Theory of Planned Behaviour, could be considered (39, 40).

A key strength of this study was the application of an implementation science framework in guiding our study design and data collection, which allowed a comprehensive understanding of the anticipated barriers and facilitators predicting the implementation outcomes (10). In addition, early stakeholder engagement created opportunities for us to develop and/or refine implementation strategies that are relevant and contextualized to the implementation setting and implementers prior to implementation. Another strength was applying multiple techniques iteratively to accelerate data analysis processes. Applying a combination of techniques helped to support the rapidity of the analysis without sacrificing the study rigor and robustness of the approach. In addition, we managed to arrive at a higher level of interpretation with plausible links and relationships between constructs. While the rapid qualitative approach facilitated timely feedback to implementers, we found that the intensity required with the use of a combination of techniques was high. Extensive preparatory work prior to data collection, along with targeted planning, iterative and frequent team discussions during data collection and analysis, and the involvement of experienced researchers familiar with qualitative research approaches, subject matter expertise, and implementation frameworks and theories, were required to facilitate the rapidity while keeping the rigor of the research. Thus, a rapid approach might not be suitable among teams with newly trained researchers having less experience and familiarity with the field or topic studied. This finding is similar to the literature that highlighted the reliance on experienced researchers to obtain the same quality of findings as when using conventional analysis techniques as the potential challenge in applying rapid techniques (20).

There were a few limitations. First, we did not include the perspectives of older adults, which limited our ability to identify barriers specific to them and might potentially affect the implementation strategy selection and development. However, we will engage older adults in the subsequent pilot phase after they have experienced the innovation for at least 6 months. Second, we directly coded from audiotapes, which might lead to a potential loss of data during analysis. However, this was substantially mitigated by having two coders and a reliability check against two transcripts. Third, we used a table-based matrix to summarize and communicate key points of our findings, which might lead to a loss of nuance, richness, and depth of the data presented. However, this was mitigated using multiple tools and techniques in data synthesis, iteration between team members, and participants' validation to ensure we captured the relevant points in the summary. While we sought to include participants with prior experience in community screening and complex care, the INFINITY-ICOPE care model remains novel. It cannot be ascertained whether their experience could have positively or negatively influenced the perceptions. Future research could focus on teasing out mechanisms of changes, determinants of individual behavior, and factors affecting sustainment.

We will use the findings from this pre-implementation phase to inform subsequent phases on the co-development of strategies for INFINITY-ICOPE implementation. The CFIR-identified barriers will be mapped to implementation strategies using the CFIR-ERIC mapping tools (41), followed by prioritization, selection, specification, and development of implementation strategies that will be formulated collaboratively with a stakeholder panel.

## 5 Conclusions

In this pre-implementation phase, we focused on adoptability as the key anticipated implementation outcome and assessed the key influences predicting this. The findings demonstrated agreement toward the innovation and suggested implementation readiness at clinical and service levels. However, barriers related to innovation complexity, workforce capacity and capability, and incompatibility with older adults' characteristics should be addressed. Adoptability likely depends on intrinsic and extrinsic motivating factors, alignment with priorities, and the minimization of tradeoffs by providing sufficient support systems and collaborative networks to implementers. The CFIR-identified barriers and enablers will guide the mapping, selection, specification, and development of implementation strategies (41–43).

## Data Availability

The datasets presented in this article are not readily available because we wish to protect the privacy rights of the participants in accordance with the informed consent of this study. Requests to access the datasets should be directed to Mimaika Luluina Ginting, ginting.mimaika.luluina@geri.com.sg.
