# Supplementary material for: Integrated frailty and intrinsic capacity care model for community-dwelling older adults in Singapore: a rapid qualitative study of anticipated implementation barriers and enablers using the Consolidated Framework for Implementation Research and its Outcomes Addendum
Source: Front Health Serv. 2025 Apr 24;5:1563686. doi: 10.3389/frhs.2025.1563686 (PMC12058744; doi:10.3389/frhs.2025.1563686)
Supplement: Supplementary file 1 [file Datasheet1.pdf]

Supplementary Data 1. Example of Individual RREAL sheet

Study participants: Clinicians group
Type of session: Focus Group Discussion

Topic	Key points
**Perception about INFINITY-ICOPE (perceived agreement and acceptability of the innovation)**	• Perceived agreement and acceptability of the INFINITY-ICOPE (the innovation). It is perceived as a sound and logical approach based on very strong evidence/guidelines from the World Health Organisation (WHO).<br><br>• Perceived agreement with the innovation design, including direct referral access and collaborative nature of work with specialised care e.g., direct neuroimaging access.<br><br>• Perceived importance of needs-based discharge criteria (as assessed by the multidisciplinary team), as opposed to discharging patients based on a time-limited service.
**Relative advantage of INFINITY-ICOPE as compared to other similar programs or alternatives**	The INFINITY-ICOPE care model is novel, very grounded in evidence and much more comprehensive, compared to similar ongoing programs in their respective current practice settings.<br><br>There are perceived advantages in:<br>• Having comprehensive steps for screening, needs assessment, care planning, referral, and care management that targets multi-domains of care.<br>• The availability of and ability to tap into a multi-disciplinary care team, which is perceived as a good approach for frailty and for patients who need complex care.<br>• Enabling the care for frail older adults in the community setting.
**Appropriateness of INFINITY-ICOPE in supporting and optimizing functional ability of older adults**	The INFINITY-ICOPE is perceived as an appropriate care model for targeting frailty and individuals who require complex care.<br><br>There are perceived potential barriers that limit the compatibility of the innovation with practice settings:<br><br>• There are differences between the criteria of the target population for the INFINITY-ICOPE with the profile of current clients seen in the practice setting. For example, patients in the existing programme for frail older adults and memory clinic are older and enrolled from specific sources. The INFINITY-ICOPE may have a wider outreach of older persons who are younger (from 60 years old) and from activity centre for older adults. There may potentially be a mismatch between the manpower/resources in the practice setting and the volume of clients.

	• With the implementation of INFINITY-ICOPE, there may be different programs/functions in the practice setting. Each program may have target populations, care functions, and care delivery pathways that are different or overlap. Thus, there is uncertainty on how the various programs will be synchronised and operationalised.
**Feasibility to implement INFINITY-ICOPE**	There are perceived potential barriers and facilitators to implementing the INFINITY-ICOPE, both at the practice level and individual level (involving both innovation deliverers and recipients):<br><br><u>Practice level</u><br>• Limited availability of infrastructure i.e., space/room to cater for high volume/reach of target population, both at public and private setting.<br><br>• Unavailability of transport arrangements to support clients with barriers to transportation. Lack of transport arrangements and the need to travel to multiple places could be a barrier to patients.<br><br>• Limited resources in manpower:<br>o Limited manpower (quantity) available to cater for wider reach of target population and to provide needs-based care (as opposed to a time-limited service)<br>o Limited manpower (quality) trained for geriatric care<br>o Limited resources available to train (new) providers<br>o High turnover rate in public setting might affect the ability of the practice setting to implement the innovation<br><br>• Availability of sufficient funding to provide required resources (i.e., space, manpower) is perceived as a facilitator to implementation.<br><br>• Clarity on and perceived fairness of the incentive system to remunerate for the allocated workload is perceived as facilitator to implementation.<br><br><u>Individual level – innovation deliverers</u><br>• There is a perceived gap in the capability of the potential healthcare providers involved in the implementation:<br>o Healthcare providers may not understand the concept of frailty<br>o There is a perception that there are different levels of competencies in providing geriatric care across different sites/Some staff may not be trained at all in delivering geriatric care.<br>o Perceived uncertainty / differences in the competency / confidence of the nurses to carry out upstream processes well.

<table>
<tr><td></td><td>
<li style="list-style:none">
Perceived anxiety and lack of confidence from new providers involved in the care delivery due the gap in knowledge and/or skills.
There are perceived barriers to opportunity for capability building:
Limited time and resources (experts, space) to train and teach new healthcare providers.
There is a perception that there is of less direct supervision for later adopters.
Inability to block time to attend training due to work schedule.
Capability building could be facilitated by good and sufficient access to knowledge and information (or training). Some suggestions to facilitate capability building:
Direct one-to-one supervision and on-the-job training from implementation leads/facilitators.
Training on practical clinical skills (e.g., assessing swallowing).
Providing access to protocols/guidelines for care pathways.
Perceived need to have more collaborative work between private and public healthcare providers e.g., shared resources especially for nurses. There are uncertainties on whether there can be synergy between INFINITY-ICOPE with the current HealthierSG policy (where private GPs are included in the primary care network).
<u>Individual level – innovation recipients</u>
A potential barrier is the inability of clients to use the technology involved in the program delivery (e.g., mobile app).
A potential barrier is the inability to afford the out-of-pocket cost.
The need to go to multiple places for the initial screening and problem identification might be seen as too complex (might be either from clients’ perspective, or also from care providers). This might pose as a barrier to clients.
</td></tr>
<tr><td>Willingness to adopt INFINITY -ICOPE</td><td>
Facilitators to adoption of INFINITY-ICOPE are perceived to be available both at the practice level and individual level (involving both innovation deliverers and recipients):

<u>Practice level</u>
Time is needed to stabilise workflows and test the feasibility of the care model. The ability to start with smaller scope first (narrower target population) with staggered implementation will help (due to the limited capacity of some settings). This is perceived as facilitator to adoption.
</td></tr>
</table>

	• The ability to pilot test the program is perceived to facilitate willingness to adopt.<br><br>Individual level – innovation deliverers<br>• Motivation to adopt is influenced by:<br>o Top-down decision (mostly for public setting).<br>o Having more time allocated for care delivery.<br>o Internal motivation to serve patients as per role.<br>o Clear incentive and remuneration mechanism.<br><br>Individual level – innovation recipients<br>• There is a limited ability to understand the importance, benefit or needs of the innovation, and lack of awareness or understanding of the concept (e.g., on preventive health or frailty). Frailty or deficit in intrinsic capacity might be perceived as a normal part of ageing by clients., Hence, the perceived need for and, benefit of the care model and management are not there.<br><br>• Limited capability of the recipients might influence their motivation to adopt the innovation and to comply with the recommended care pathways (e.g., to follow-up with the recommended care management). It could also affect their willingness to pay for the innovation.<br><br>• Increasing the awareness and knowledge of the benefit and importance of innovation via tailored messaging will facilitate adoption by potential recipients/caregivers.<br><br>• Engagement and education to older adults and their primary caregivers on the importance and benefits of care model (and the expected steps) must be communicated early (more upstream in the care model) with the right approach or tailored messaging. Understanding the family dynamics is also important in communication with patients.<br><br>• Involvement of other family members as well (eg children), because the primary caregiver (eg elderly spouse) may not have the necessary capability or position to make decisions on behalf of the recipient.<br><br>• Duplication of similar programs in the community with the same target population might confuse the recipients (e.g., HealthierSG)
**Suggestions for adaptation of INFINITY-ICOPE**	Having multidisciplinary team, Comprehensive Geriatric Assessment (CGA), and referral pathways to specialised care are perceived as important components of the care model (that should not be changed).

	Simplification/changes to the care model that could be considered:<br>• To have the initial screening and problem identification steps at the same site<br>• To provide alternatives for non-tech-savvy participants e.g., providing iPads to participants at the site for them to submit data for initial screening and re-assessment.<br>• To empower nurses to do referrals directly to specialised care for certain isssues that can be identified at an early phase (without having to go through primary care physician such as in Step 2). This might be supported with a standardised protocol.
