# Supplementary material for: Integrated frailty and intrinsic capacity care model for community-dwelling older adults in Singapore: a rapid qualitative study of anticipated implementation barriers and enablers using the Consolidated Framework for Implementation Research and its Outcomes Addendum
Source: Front Health Serv. 2025 Apr 24;5:1563686. doi: 10.3389/frhs.2025.1563686 (PMC12058744; doi:10.3389/frhs.2025.1563686)
Supplement: Supplementary file 2 [file Image1.pdf]

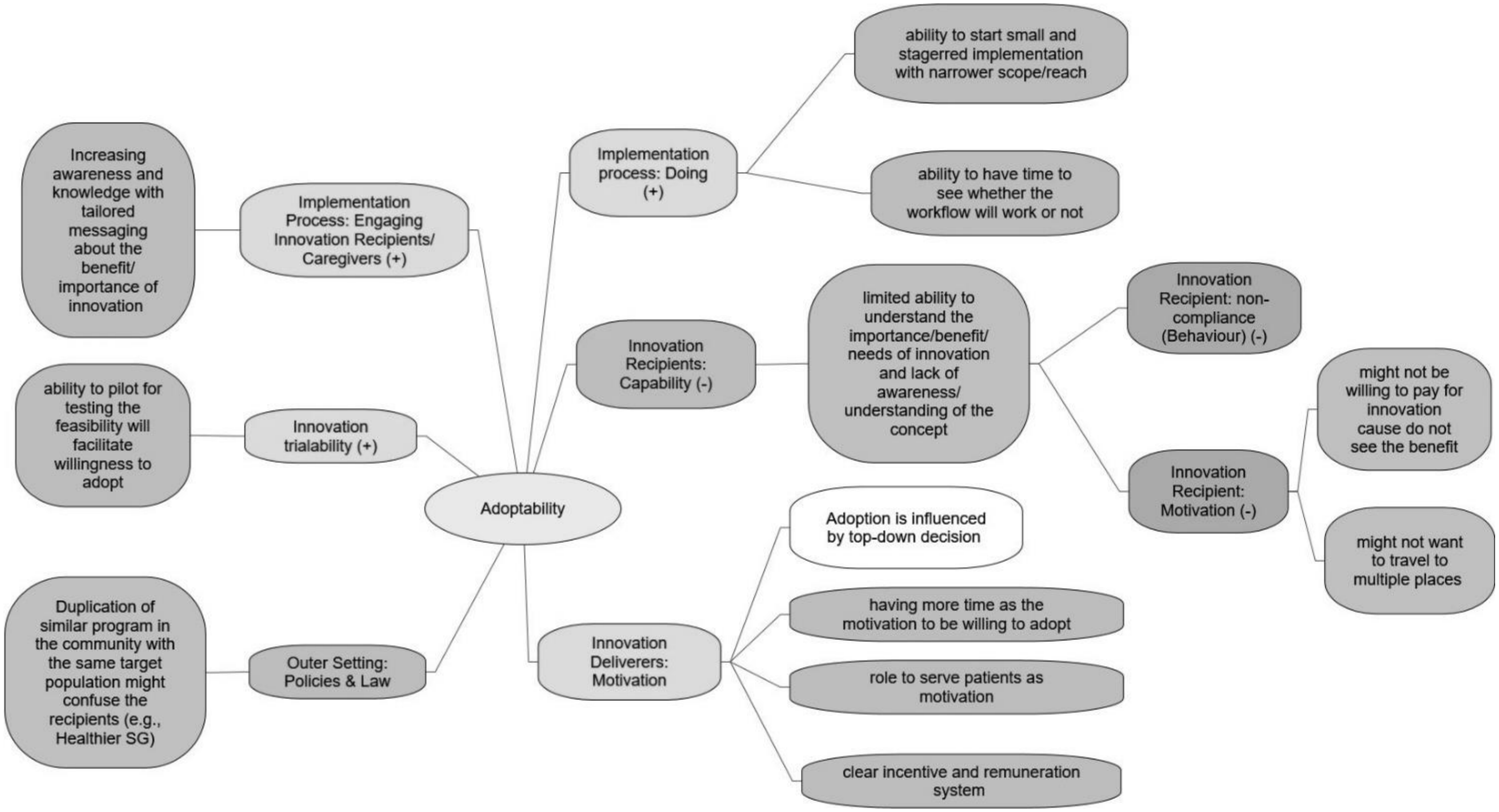

Supplementary Figure 1. Example of mind-map

CFIR: Consolidated Framework for Implementation Research

(+) CFIR construct was an enabler and/or there was a positive mention of CFIR construct in influencing the likelihood to adopt the INFINITY-ICOPE care model

(-) CFIR construct was a barrier and/or there was a negative mention of construct in influencing the likelihood to adopt the INFINITY-ICOPE care model
