# Supplementary material for: Integrated frailty and intrinsic capacity care model for community-dwelling older adults in Singapore: a rapid qualitative study of anticipated implementation barriers and enablers using the Consolidated Framework for Implementation Research and its Outcomes Addendum
Source: Front Health Serv. 2025 Apr 24;5:1563686. doi: 10.3389/frhs.2025.1563686 (PMC12058744; doi:10.3389/frhs.2025.1563686)
Supplement: Supplementary file 3 [file Table1.pdf]

**Supplementary Table 1.** Standards for Reporting Qualitative Research (SRQR) checklist

| No | Topic | Item description | |
|---|---|---|---|
| **Title and abstract** | | | |
| S1 | Title | Concise description of the nature and topic of the study Identifying the study as qualitative or indicating the approach (e.g., ethnography, grounded theory) or data collection methods (e.g., interview, focus group) is recommended | Refer to study title.<br><br>*Integrated frailty and intrinsic capacity care model for community-dwelling older adults in Singapore: a rapid qualitative study of anticipated implementation barriers and enablers using the Consolidated Framework for Implementation Research and its Outcomes Addendum* |
| S2 | Abstract | Summary of key elements of the study using the abstract format of the intended publication; typically includes background, purpose, methods, results, and conclusions | Refer to Abstract. |
| **Introduction** | | | |
| S3 | Problem formulation | Description and significance of the problem/phenomenon studied; review of relevant theory and empirical work; problem statement | Refer to *Introduction* section in the main text. |
| S4 | Purpose or research question | Purpose of the study and specific objectives or questions | Refer to *Materials and Methods* section, sub-sections: *Study aims and design.* |
| **Methods** | | | |
| S5 | Qualitative approach and research paradigm | Qualitative approach (e.g., ethnography, grounded theory, case study, phenomenology, narrative research) and guiding theory if appropriate; identifying the research paradigm (e.g., postpositivist, constructivist/ interpretivist) is also recommended; rationale | Refer to *Materials and Methods* section, sub-sections: *Study aims and design.* |
| S6 | Researcher characteristics and reflexivity | Researchers' characteristics that may influence the research, including personal attributes, qualifications/experience, relationship with | Refer to *Materials and Methods* section, sub-section: *Data collection.* |

| | | | |
|---|---|---|---|
| | | participants, assumptions, and/or presuppositions; potential or actual interaction between researchers' characteristics and the research questions, approach, methods, results, and/or transferability | |
| S7 | Context | Setting/site and salient contextual factors; rationale | Refer to *Materials and Methods* section, sub-section: *The innovation* |
| S8 | Sampling strategy | How and why research participants, documents, or events were selected; criteria for deciding when no further sampling was necessary (e.g., sampling saturation); rationale | Refer to *Materials and Methods* section, sub-section: *Sampling* |
| S9 | Ethical issues pertaining to human subjects | Documentation of approval by an appropriate ethics review board and participant consent, or explanation for lack thereof; other confidentiality and data security issues | Refer to *Materials and Methods* section, sub-section: *Study aims and design* |
| S10 | Data collection methods | Types of data collected; details of data collection procedures including (as appropriate) start and stop dates of data collection and analysis, iterative process, triangulation of sources/methods, and modification of procedures in response to evolving study findings; rationale | Refer to *Materials and Methods* section, sub-section: *Data collection* |
| S11 | Data collection instruments and technologies | Description of instruments (e.g., interview guides, questionnaires) and devices (e.g., audio recorders) used for data collection; if/how the instrument(s) changed over the course of the study | Refer to *Materials and Methods* section, sub-section: *Data collection* |
| S12 | Units of study | Number and relevant characteristics of participants, documents, or events included in the study; level of participation (could be reported in results) | Refer to *Results* section, sub-section: *Characteristics of participants (Table 1)* |
| S13 | Data processing | Methods for processing data prior to and during analysis, including transcription, data entry, data management and security, verification of data integrity, data coding, and anonymization/deidentification of excerpts | Refer to *Materials and Methods* section, sub-section: *Data analysis* |

| | | | |
|---|---|---|---|
| S14 | Data analysis | Process by which inferences, themes, etc., were identified and developed, including the researchers involved in data analysis; usually references a specific paradigm or approach; rationale | Refer to *Materials and Methods* section, sub-section: *Data analysis* |
| **Results/findings** | | | |
| S16 | Synthesis and interpretation | Main findings (e.g., interpretations, inferences, and themes); might include development of a theory or model, or integration with prior research or theory | Refer to *Results* and *Discussions* section. |
| S17 | Links to empirical data | Evidence (e.g., quotes, field notes, text excerpts, photographs) to substantiate analytic findings | Refer to *Supplementary materials (examples from RITA coding, mind-map, and individual RREAL sheet)* |
| **Discussion** | | | |
| S18 | Integration with prior work, implications, transferability, and contribution(s) to the field | Short summary of main findings; explanation of how findings and conclusions connect to, support, elaborate on, or challenge conclusions of earlier scholarship; discussion of scope of application/ generalizability; identification of unique contribution(s) to scholarship in a discipline or field | Refer to *Discussions and Conclusion* sections |
| S19 | Limitations | Trustworthiness and limitations of findings | Refer to *Materials and Methods* section, sub-section: *Data analysis;* and *Discussion* section |
| **Other** | | | |
| S20 | Conflicts of interest | Potential sources of influence or perceived influence on study conduct and conclusions; how these were managed | Refer to *Declarations* section, sub-section: *Conflict of interest* |
| S21 | Funding | Sources of funding and other support; role of funders in data collection, interpretation, and reporting | Refer to *Declarations* section, sub-section: *Funding* |
