# Supplementary material for: Integrated frailty and intrinsic capacity care model for community-dwelling older adults in Singapore: a rapid qualitative study of anticipated implementation barriers and enablers using the Consolidated Framework for Implementation Research and its Outcomes Addendum
Source: Front Health Serv. 2025 Apr 24;5:1563686. doi: 10.3389/frhs.2025.1563686 (PMC12058744; doi:10.3389/frhs.2025.1563686)
Supplement: Supplementary file 4 [file Table2.docx]

**Supplementary Table 2.** Focus Group Discussion Topic Guide

| **CFIR Outcomes Addendum** | **CFIR Implementation determinants*** | **Main questions** | **Probing questions**  **(only when needed)** |
| --- | --- | --- | --- |
| **Context / Background** |  | Q1. What is your professional background and how long have you been in this role? |  |
|  |  | Q2. How is the nature of your daily work? | - Are you working with a team, or individually? - Within your organization only or with external organizations? |
| **Acceptability** | - **Innovation design**   The innovation is well designed and packaged, including how it is assembled, bundled, and presented.   - **Innovation complexity**   The innovation is complicated, which may be reflected by its scope and/or the nature and number of connections and steps.   - **Innovation relative advantage**   The innovation is better than other available innovations or current practice. | Q4. We would like to hear your thoughts about the care pathway. What do you think about:  ---the technology involved  ---the ICOPE Steps (or the sequences) – ie the screening step, each of the subsequent steps (referring to the diagram and ask each color code area)  ---how different healthcare providers need to collaborate? |  |
|  |  | Q5. How does the ICOPE compare to other similar existing programs or alternatives that may have been considered in your organization (or centre)? | - Is there any? |
| **Appropriateness** | - **Compatibility**   The innovation fits with workflows, systems, and processes   - **Mission alignment**   Implementing and delivering the innovation is in line with the overarching commitment, purpose, or goals   - **Relative priority**   Implementing and delivering the innovation is important compared to other initiatives (in relation to organization priority i.e., whether there is a strong sense of the importance of the program as key organizational priority)   - **Characteristics: Need**   The individual(s) has deficits related to survival, well-being, or personal fulfilment, which will be addressed by implementation and/or delivery of the innovation | Q6. In what way do you think ICOPE could address the issue on preventing and reducing functional decline in older people? |  |
|  |  | Q7. How appropriate do you think it is to implement this program in your setting? | - Does ICOPE fit with what you and/or your organization is doing? - Does it **align with the organization** performance measures? - Does it **align with your** vision/values? |
| **Feasibility** | - **Available resources** (funding, physical space, materials & equipment, etc.)   Resources are available to implement and deliver the innovation.   - **Access to Knowledge & Information**   Guidance and/or training is accessible to implement and deliver the innovation   - **Structural characteristics** (physical infrastructure, information technology infrastructure, work infrastructure, etc.)   Infrastructure components support functional performance of the Inner Setting   - **Innovation Cost**   The innovation purchase and operating costs are affordable   - **Partnerships & Connections**   The Inner Setting is networked with external entities, including referral networks, academic affiliations, and professional organization networks   - **Relational connections**   There are high quality formal and informal relationships, networks, and teams within and across Inner Setting boundaries (e.g., structural, professional)   - **Communications**   There are high quality formal and informal information sharing practices within and across Inner Setting boundaries (e.g., structural, professional)   - **Culture**   There are shared values, beliefs, and norms across the Inner Setting | Q8. What kind of support or resources would your organization need, if you were to implement the ICOPE? | - To probe for the support/resources needed for each steps i.e.,   --Screening,  --Comprehensive needs assessment,  --Individualized care development,  --Care coordination and referral with other community and/or primary care settings   - - Is there a formal linkage/coordination? How does a referral work?   - Experience when referring older adults for care services in other community and/or primary care setting (e.g., referral process to CNPs or GSH, referral to community care services) - Support / resources needed **as an individual** doing your professional role (e.g. training, time/availability, work arrangement, skills, knowledge, competency) - Support / resources **as an organization** (e.g., physical infrastructure, IT infrastructure, funding, materials, equipment) |
|  |  | Q9. Do you think your current organization (or centre) has sufficient resources or support needed to implement ICOPE? How do you think this could be addressed? |  |
| **Adoptability** | - **Characteristics: Capability, Opportunity, Motivation**   Has interpersonal competence, knowledge, and skills to  fulfill role; has availability, scope and power to fulfil role; is committed to fulfilling role.   - **External pressure: Performance-measurement pressure**   Quality or benchmarking metrics or established service goals drive implementation and/or delivery of the innovation | Q10. Would you (or your organization) be willing to implement (or adopt) the ICOPE? | - What would motivate you/your organization to implement ICOPE? - What would hinder you/your organization to implement ICOPE? |
| **Appropriateness** | - **Innovation adaptability**   The innovation can be modified, tailored, or refined to fit local context or needs | Q11. What kind of changes (adaptation) do you recommend for the program to be implemented well in your setting? | Are there components that can be simplified and/or removed, or included? Why?  Which parts should not be changed, and which parts could be adapted?  What are your recommendations on revising the program that will enable you to adopt it? |

*List of selected CFIR determinants that might potentially influence the antecedent assessments or outcome, to aid further probing. The list is not exhaustive.
