# Supplementary material for: Integrated frailty and intrinsic capacity care model for community-dwelling older adults in Singapore: a rapid qualitative study of anticipated implementation barriers and enablers using the Consolidated Framework for Implementation Research and its Outcomes Addendum
Source: Front Health Serv. 2025 Apr 24;5:1563686. doi: 10.3389/frhs.2025.1563686 (PMC12058744; doi:10.3389/frhs.2025.1563686)
Supplement: Supplementary file 5 [file Table3.docx]

**Supplementary Table 3**. Example of Rapid Identification of Themes from Audio recordings (RITA) coding

| **Time stamp (minutes)** | 0 - 5 | 5 - 10 | 10 - 15 | 15 - 20 | 20 - 25 | 25 - 30 | 30 - 35 | 35 - 40 | 40 - 45 | 45 - 50 | 50 - 55 | 55 - 60 | 60 - 65 | 65 - 70 |
| --- | --- | --- | --- | --- | --- | --- | --- | --- | --- | --- | --- | --- | --- | --- |
| **Code** |  |  |  |  |  |  |  |  |  |  |  |  |  |  |
| **Innovation design** |  |  |  |  | (-)  unavailability of engagement/communication/messaging about the importance of preventive care, esp on frailty to older adult |  | (-)  approach for defining and screening target population is wide (60 years and above, from AAC) and reach might be overwhelmed with the capacity of public setting |  | (+)  (43:00), it is good so that we can care for frail patients in the community settings, rather than referring them straight to specialised care/hospital |  | (-)  due to the limitation of transport arrangement, physio/occupational therapy session by community partners was done via zoom - this is perceived as not effective | (-)  there are so many places to go for screening and initial assessment/identification | (+)  direct access to neuroimaging, allowing to refer to specialist as appropriate. 'should never be a case that the specialist hands off and refer back to GP and polyclinic, I think there should always be something that we can fall back on' (1:04:04) | (-)  a time limited service (like the current existing programme) doesn't feel like the best handled, ‘cause it is based on how many subsidized visits they could have within the programme. Patients that are more frail may need more time than that. |
| **Innovation relative advantage** |  |  |  | (+)  novel and screen for frailty |  |  |  |  | (+)  much more comprehensive, 'very medical', with screening, comprehensive geriatric assessment, very grounded in the evidance, as compared with others which is only driven by the need to meet a certain service reach KPI (41:00) | (+)  comparing to other program in their setting: the other program is very 'one direction', only targeting one domain (i.e., frailty), and offering exercise program, not very comprehensive (as compared to INFINITY)) |  |  |  |  |
| **Innovation Evidence Base** |  |  |  |  |  |  |  |  | (+)  very logical, based on a very strong evidence based on WHO guideline |  |  |  |  |  |
| **Innovation trialability** |  |  |  |  |  |  | (+)  pilot phase is necessary to assess feasibility |  |  | (o)  will need time to see how it works, and give more feedback | (o)  only when we are doing pilot program first (i.e., having one or two cases and refer to private sector), then we can see how it goes |  |  |  |
| **Compatibility** |  |  |  |  |  |  | (+)  available experienced resources and experts from prior experience - established centre and workflows (30:00) |  | (o)  needs clarity on who are the target audience and how to resolve the manpower issue to implement |  |  |  | (-)  there is a gap in the target population age group, currently the memory clinic only see patients 65 years and above | (o)  as this will be the expansion of the current existing program, i.e., there will be additional upstream and downstream processes, don't know how the different target age group would be operationalized |
| **Available resources: Others** |  |  |  |  |  | (+)  shared resources between private and public healthcare setting  (-)  unavailability of trained staff in geriatric care in private setting | (+)  will be able to run with nursing support - private setting  (+)  available experienced resources and experts from prior experience - established centre  (-)  not enough manpower to sit in/do training with the new doctors in multiple rooms - public | (o)  timeline relating to timing and the scope of target population (recording not too clear) (36:00) |  |  |  |  |  | (-)  more resources needed to have a non-time limited services |
| **Available resources: Funding** |  |  |  |  |  |  | (o)  more money is needed to support resources if expanding the scope of the program (more manpower, space, time for more complex cases) |  |  |  | (o)  more money needs to be poured in, for manpower e.g., clinicians, nurses, allied health | (+)  'I have faith that you guys will give us incentive..' -- on willingness to adopt if the incentive is clear; the question is how will the reimbursement will be etc. (55:23)  (o)  cost involved in implementing will need to take into account for the doctor time used (for consultation based on time allocation e.g., 15-30 mins per case), for nurses and admin works has to be factored in as well |  |  |
| **Individual Characteristics (Innovation Deliverers): Capability** |  |  |  |  | (-)  on healthcare providers and clinicians not understanding the concept of frailty | (-)  uncertainty of the confidence of nurses to do assessment and refer only high risk patients to downstream (28:00)  competency of the nurses in relation to upstream processes will affect the workload in the downstream processes - there is different level of competency across sites |  |  |  | (-)  will need support on formal training, new doctors felt anxious when facing the clients for the first time  (+)  all these trainings made the individual confident and able to run the program -- perceived the necessity of this level of resources/training to equip the doctor |  |  |  |  |
| **Individual Characteristics (Innovation Deliverers): Opportunity** |  |  |  |  |  |  |  |  |  | (-)  only manage to attend one (zoom/in-person) training due to manpower issue |  |  |  |  |
| **Individual Characteristics (innovation deliverers): Motivation** |  |  |  |  |  |  |  |  |  |  |  | (o)  do not have a say in adoption/implementation. If the roster is block at a certain time to deliver the program, will just do ' I mean, I am an employee (laugh)'; (57:10)  (+)  having more time ('it's an institution... if polyclinic say that they will support this program and they want to run this program, then they give us the resources, then we will do, have to do it lah, but i think what would make me more willing to do it is..time')  (+)  'for me...just keep serving my patients..’ |  |  |
| **Innovation Recipients: Capability** |  |  | (-)  Limitation on technology for older adult (13:32) |  |  |  |  |  |  |  |  |  |  |  |
| **Innovation Recipients: Motivation** |  |  | (-)  Older adult has limited awareness and ability to understand the innovation and the importance/  benefits of innovation (14:42) | (-)  Older adult has limited awareness and ability to understand the innovation and the importance/benefits of innovation (15:00); (18:00) |  |  |  |  |  |  | (-)  some of older adults who are not willing to pay, don't know whether they really don’t have the money or are not willing to pay, because they don't see the benefit ('not worth 10 dollars to me') | (-)  between step 1 to step 2, there might be patient who do not want to travel to the next place |  |  |
| **Innovation Recipients: Opportunity** |  |  |  |  |  |  |  |  |  |  | (+)  providing a transport service (e.g., school bus) to bring elderly to the community site, might be better  (-)  transport and traveling are another big barrier that patients face |  |  |  |
